# The impact of nontraditional lipid profiles on left ventricular geometric abnormalities in general Chinese population

**DOI:** 10.1186/s12872-018-0829-x

**Published:** 2018-05-08

**Authors:** Haoyu Wang, Zhao Li, Xiaofan Guo, Yintao Chen, Ye Chang, Shuang Chen, Yingxian Sun

**Affiliations:** Department of Cardiology, The First Hospital of China Medical University, 155 Nanjing North Street, Heping District, Shenyang, 110001 Liaoning People’s Republic of China

**Keywords:** Left ventricular geometry, Left ventricular hypertrophy, Nontraditional lipid profiles, Lipids, General population

## Abstract

**Background:**

Despite current interest in the unfavorable impact of nontraditional lipid profiles on cardiovascular disease, information regarding its relations to abnormal left ventricular (LV) geometry has not been systemically elucidated. This study sought to understand predictive implication of nontraditional lipid profiles in specific LV geometric patterns in the general population of rural China.

**Methods:**

Analyses were based upon a cross-sectional study of 10,756 participants (mean age 53.8 years; 54.0% females) who underwent assessment of biochemical, anthropometric, and blood pressure variables in rural areas of China. Participants were classified into four groups of LV morphologic pattern according to left ventricular mass index (LVMI) and relative wall thickness with quantitative echocardiographic data.

**Results:**

By multivariable-adjusted linear regression models, nontraditional lipid profiles were positive determinants of concentricity index and LV wall thickness (all *P* < 0.05), with modest effects on LVMI. Non-high-density lipoprotein cholesterol (non-HDL-C) emerged as an independent correlate of concentric LV hypertrophy (LVH) (adjusted odds ratio [OR]: 1.174 per 1 SD increment in non-HDL-C, 95% confidence interval [CI]: 1.075–1.281), followed by low-density lipoprotein cholesterol (LDL-C)/HDL-C ratio (1.158 [1.059–1.266]), total cholesterol (TC)/HDL-C ratio (1.150 [1.050–1.260]), and triglyceride (TG)/HDL-C ratio (1.134 [1.030–1.249]). The ORs for concentric LVH by tertiles further provided insight into that excess risk was associated with the highest tertile of nontraditional lipid profiles. The areas under the ROC curves to predict concentric LVH were statistically identical among nontraditional lipid parameters.

**Conclusion:**

Nontraditional lipid profiles, easily measured in the everyday routine examination, were responsible for increased risk of concentric LVH, potentially providing enhanced clinical utility at no additional cost, which emphasized the beneficial effect of these markers to supplement and improve CVD risk stratification.

## Background

Left ventricular hypertrophy (LVH), a potent marker of end-organ damage, is an independent predictor and potentially modifiable risk factor for coronary heart disease (CHD), heart failure (HF), and other major cardiovascular (CV) morbidity and mortality [1, 2]. In light of left ventricular mass index (LVMI) along with relative wall thickness (RWT), four distinct patterns of left ventricular (LV) geometry are further proposed to explain the pathophysiologic basis for cardiac remodeling, with concentric LVH conferring a stronger prognostic value of morbid CV events [3, 4]. Although greater left ventricular mass (LVM) might initially be considered beneficial by a concomitant LV wall stress reduction and avoidance of hemodynamic compromise, in long-term, the progression of LV geometric abnormalities, particularly LVH, can prove to be maladaptive and portend a poor prognosis [5–7]. Identification and intervention of the CV risk factors underlying a specific phenotype of LV geometry before development of manifest disease are of paramount importance, given that knowledge of these geometric adaptations has been a fundamental precursor of CV disorders.

Reports from observational studies on the possibility of dyslipidemia-induced LV remodeling have been inconsistent [8–16]. Several lines of evidence have demonstrated that low high-density lipoprotein cholesterol (HDL-C) may unfavorably modify LV structure in the setting of elevated LVM or presence of LVH [8–10], whereas others have not [11–13]. Historically, there were conflicting data as to whether greater total cholesterol (TC) has been implicated in increased LVM [15, 16]. It was also plausible that alterations in LV morphology would be related at least in part to the excess of triglyceride (TG) which favors a high LVM-to-volume ratio and LV end-diastolic volume [12]. Conversely, lipid derangements, represented as cholesterol remnants and TG, were insufficient to cause an overt increase in LVM and LV wall thickness [16]. Recent emphasis has been placed in the clinical implications of nontraditional lipid profiles as powerful and independent predictors of cardiovascular disease (CVD) outcomes [17–21]. For instance, previous studies revealed that TC/HDL-C ratio could represent a simple atherogenic particle burden tool informing on lipoprotein particle concentration and size not available in cholesterol-based measurements [22, 23]. Notably, TC/HDL-C ratio offered significant incremental prognostic information over low-density lipoprotein cholesterol (LDL-C) and non-HDL-C for predicting CVD events [18, 20, 24]. Moreover, TG/HDL-C ratio has received growing interest in identifying insulin resistance and concentrations of sd-LDL particles as a novel, inexpensive, and readily available biomarker for quantifying atherogenic and cardiometabolic risk [17, 19, 25, 26]. Further, assessment of lipid metabolism and atherosclerotic status using a simple tool, such as LDL-C/HDL-C ratio, not only could help closely reflecting the interactions between lipid fractions but also could better predict plasma atherogenicity than isolated lipid values [18, 27, 28]. It has recently been proposed that non-HDL-C that includes all of the atherogenic lipoproteins, such as TG-rich lipoproteins, intermediate-density lipoprotein cholesterol, LDL-C, and lipoprotein(a) could better predict cardiovascular outcomes in patients on LDL-C-lowering therapy [20, 24].

To date, data are sparse regarding the relative performance of nontraditional lipid profiles for the purpose of reclassification of LV geometry risk. Di Bonito P et al. advocated that a high TG/HDL-C ratio, independent of blood pressure and visceral adiposity, was a key determinant of worrisome concentric LVH [29]. Interestingly, an epidemiological investigation from the Framingham Heart Study did not support an independent correlation of non-HDL-C and LV remodeling [11]. In this regard, it is likely that the adverse impacts of nontraditional lipid profiles on CVD incidence may partly be mediated by it adverse effects on LV structural remodeling. However, no prior work has examined this premise comprehensively. We hypothesized that evaluating the influence of nontraditional lipid profiles on the probability of subclinical LV geometry change is helpful to understand the pathogenesis of CVD related to dyslipidemia. Accordingly, the current study was initiated to examine the contribution of nontraditional lipid profiles (TC/HDL-C, TG/HDL-C, LDL-C/HDL-C, and non-HDL-C) to the prevalence of morphologic LV abnormalities in the general population of rural China.

## Methods

### Study population

This study population was identified from Northeast China Rural Cardiovascular Health Study (NCRCHS). The NCRCHS study was a population-based cross-sectional epidemiological investigation evaluating the presence of cardiovascular risk factors in 11,956 permanent residents (≥ 35 years of age) of northeast China from January 2013 to August 2013. Details regarding the rationale, design, and implementation have been described previously [30–32]. Each participant provided informed consent, and institutional review board of China Medical University approved study protocols. After further excluding subjects with lipid-lowering medication use (*n* = 371) and missing serum lipoproteins and echocardiographic data (*n* = 829), 10,756 individuals were included.

### Data collection and measurements

Of relevance to the current analysis, the demographic socioeconomic factors, healthy habits, and comprehensive medical history were gathered from medical records and patients interviews, collected by standardized questionnaires. Data collected during the clinic examination included weight, height, and body mass index (BMI), blood pressure (BP), fasting plasma glucose (FPG), and lipid fractions. The existence of history of CVD (coronary heart disease, arrhythmia and heart failure) were defined as any self-reported previous physician diagnosis. Individuals were considered to be taking antihypertensive, antidiabetic, and lipid-lowering medication based on self-reported use within the past 2 weeks of the baseline interview. A full description of data collection and methods selection of this sample has been already published [30–32].

In brief, standing height was measured using a wall-mounted stadiometer; body weight was measured on a calibrated digital scale. These measurements were used to calculate BMI as measured weight in kilograms divided by standing height in meters squared. Seated BP was measured by a standardized automatic electronic sphygmomanometer (HEM-907; Omron, Kyoto, Japan) using an appropriately sized cuff with the arm supported at the level of the heart. Each subject sat quietly with legs uncrossed for 5 min before BP measurement. Three consecutive BP readings were taken at least 5 min apart, and the average of the last 2 measurements was recorded for analysis. FPG, TC, TG, LDL-C, and HDL-C were obtained on fasting blood samples (> 12 h). Non-HDL-C was calculated as the difference between TC and HDL-C. All the blood measurements were followed the same protocol.

### Echocardiography

The designs of the echocardiographic protocols for the NCRCHS study were described previously. [31, 32]. M-mode measurements, averaged over ≥3 cardiac cycles, were obtained from digitized images for interventricular septal thickness (IVST), posterior wall thickness (PWT), and LV internal diameter in end diastole (LVIDD) and systole at the time of the index examination (in accordance with the American Society of Echocardiography [ASE] guidelines) [33]. We used the following formula described by Devereux [34] to calculate the unadjusted LVM: LVM = 0.8 × [1.04 × ((IVST + PWT + LVIDD)^3^–LVIDD^3^)] + 0.6. The sum of IVST and PWT was used as an estimate of left ventricular wall thickness (LVWT). RWT was calculated by multiplying 2 times PWT divided by LVIDD. LVMI was calculated by dividing the LVM by height^2.7^. We used the biplane method of disks in the apical four-chamber view to calculate LV end-diastolic volume (LVEDV) AND LV end-systolic volume (LVESV). LV ejection fraction (LVEF) was computed by (LVEDV-LVESV)/LVEDV × 100%. The ratio of LVM to LVEDV determined LV concentricity index. LVH was classified as increased LVMI using thresholds of ≥48 g/m^2.7^ for males and ≥ 44 g/m^2.7^ for females [35]. Using ASE cut points for normal LVMI (< 48 g/m^2.7^ for males and < 44 g/m^2.7^ for females) and normal RWT cut-point of ≤0.42 [35], geometric patterns were defined as: normal geometry (normal LVMI and RWT), eccentric LVH (LVH and normal RWT), concentric LV remodeling (normal LVMI and increased RWT), and concentric LVH (LVH and increased RWT).

### Definition

The diagnosis of hypertension was established as blood pressure (BP) level of at least 140/90 mmHg, individuals who were on antihypertensive medications or a prior diagnosis of hypertension. The definition of diabetes was determined as follows: fasting glucose ≥7.0 mmol/L, self-reported medical diagnosis history or the current use of any blood glucose-lowering medication. Based on the recommendation for Asians [36], obesity was defined as BMI ≥ 28 kg/m^2^.

### Statistical analyses

Descriptive statistics for all covariates are summarized as mean ± standard deviation or median (interquartile range) and as frequencies (percentages) with four categories of LV geometric patterns. Demographic status, clinical characteristics, and echocardiographic parameters were compared among the different LV geometry types using ANOVA analysis with Bonferroni correction in case of multiple comparisons and chi-squared test for continuous and categorical variables, respectively. Data sets that did not have equal variances were analyzed using the nonparametric Kruskal-Wallis test. TG/HDL-C was logarithmically transformed to normalize their skewed distributions before entering into the regression analysis. The Standardized β coefficient represents the change in parameters of LV structure per 1 SD increase in nontraditional lipid profile by linear regression models. Model 1 was unadjusted. The variables of the adjusted model 2 were age, sex, race, education level, family income, diet score, smoking and drinking status, physical activity, obesity, hypertension, diabetes, history of CVD, and antihypertensive and antidiabetic medication use. For normal LV geometry as the reference, the odds ratios (ORs) and 95% confidence intervals (CIs) for independent association of per 1 SD increase in nontraditional lipid profile with abnormal LV geometry were estimated by logistic regression analyses model in a stepwise fashion. In addition, individuals were stratified into tertiles in accordance with the distribution of nontraditional lipid profiles for the purpose of estimating unadjusted and multivariable-adjusted ORs of LV geometric abnormalities. We calculated the area under the curve (AUC) by receiver operating characteristic analysis to evaluate the diagnostic ability of nontraditional lipid profiles for the identification of abnormal LV geometry. All of the statistical analyses involved the application of SPSS 22.0 software (IBM Corp) and MedCalc version 12.1.4.0 (MedCalc software, Belgium), and a two-tailed *P* < 0.05 was adopted to be statistically significant.

## Results

The age of our 10,756 participants was 53.80 ± 10.58 years, with 54.0% females. The majority of subjects had normal LV geometry (75.2%), followed by eccentric LVH (13.7%), and similar frequencies of concentric LV remodeling (6.3%) and concentric LVH (4.7%). Participants with LVH were older, less physically active, and showed a worse lipid profile (high levels of TC/HDL-C, TG/HDL-C, LDL-C/HDL-C, and non-HDL-C) compared with normal LV geometry (Table 1). Significant differences among the four groups, especially in co-morbidities (hypertension, diabetes, and obesity), were largely driven by the differences between those with and without LVH. Antihypertensive medication use and history of CVD was highest in concentric LVH. With regard to the echocardiographic variables, there were expected unfavorable outcomes in concentric LVH as opposed to eccentric LVH, characterized by higher IVST, PWT, LVWT, RWT, and LVM. The LVMI increased from normal LV geometry to concentric LVH. The LVIDD, LVEDVI and LVESVI were significantly lower in individuals with concentric LV remodeling and higher in patients with eccentric LVH.

**Table 1:** Demographic, clinical, and echocardiographic characteristics of study population according to the left ventricular geometry

Variables	Normal geometry (*N* = 8090)	Concentric LV remodeling (*N* = 681)	Eccentric LVH (*N* = 1476)	Concentric LVH (*N* = 509)	*P* value*
Age (years)	52.05 ± 10.12	57.21 ± 11.08^a^	58.53 ± 10.28^a.b^	59.85 ± 10.61^a,b^	< 0.001
Female (%)	4173 (51.6)	402 (59.0)^a^	956 (64.8)^a^	273 (53.6)^c^	< 0.001
Race (Han) (%)	7664 (94.7)	655 (96.2)	1421 (96.3)	478 (93.9)	0.023
Primary school or below (%)	3475 (46.3)	375 (55.1)^a^	948 (64.2)^a,b^	333 (65.4)^a,b^	< 0.001
Family income > 20,000 CNY/year (%)	2819 (34.8)	181 (26.6)^a^	412 (27.9)^a^	140 (27.5)^a^	< 0.001
Diet score	2.38 ± 1.12	2.20 ± 1.14^a^	2.12 ± 1.13^a^	2.22 ± 1.08^a^	< 0.001
Current smoker (%)	2971 (36.7)	230 (33.8)	426 (28.9)^a^	184 (36.1)^c^	< 0.001
Current drinker (%)	1939 (24.0)	136 (20.0)	241 (16.3)^a^	123 (24.2)^c^	< 0.001
Low physical activity (%)	2197 (27.2)	232 (34.1)^a^	538 (36.4)^a^	197 (38.7)^a^	< 0.001
Systolic blood pressure (mmHg)	137.08 ± 20.58	145.04 ± 25.34^a^	153.97 ± 24.91^a,b^	166.47 ± 25.53^a,b,c^	< 0.001
Diastolic blood pressure (mmHg)	80.45 ± 10.75	83.14 ± 12.45^a^	85.43 ± 12.81^a,b^	91.86 ± 14.15^a,b,c^	< 0.001
Fasting plasma glucose (mmol/L)	5.79 ± 1.44	6.05 ± 1.93^a^	6.13 ± 1.96^a^	6.38 ± 2.16^a,b,c^	< 0.001
BMI (kg/m^2^)	24.30 ± 3.33	23.79 ± 3.66^a^	26.69 ± 4.11^a,b^	26.51 ± 4.14^a,b^	< 0.001
TC (mmol/L)	5.16 ± 1.04	5.36 ± 1.10^a^	5.41 ± 1.16^a^	5.62 ± 1.15^a,b,c^	< 0.001
TG (mmol/L)	1.17 (0.84–1.76)	1.30 (0.92–2.01)^a^	1.45 (1.01–2.09)^a,b^	1.53 (1.07–2.40)^a,b^	< 0.001
LDL-C (mmol/L)	2.87 ± 0.79	2.98 ± 0.82^a^	3.05 ± 0.87^a^	3.25 ± 0.92^a,b,c^	< 0.001
HDL-C (mmol/L)	1.42 ± 0.38	1.45 ± 0.40	1.37 ± 0.36^a,b^	1.38 ± 0.37^b^	< 0.001
TC/HDL-C ratio	3.83 ± 1.08	3.89 ± 1.03	4.12 ± 1.09^a,b^	4.27 ± 1.15^a,b,c^	< 0.001
TG/HDL-C ratio	0.86 (0.56–1.43)	0.95 (0.63–1.59)^a^	1.11 (0.69–1.77)^a,b^	1.16 (0.74–2.08)^a,b^	< 0.001
LDL-C/HDL-C ratio	2.15 ± 0.76	2.18 ± 0.74	2.34 ± 0.80^a,b^	2.48 ± 0.83^a,b,c^	< 0.001
Non-HDL-C (mmol/L)	3.74 ± 1.01	3.92 ± 1.06^a^	4.04 ± 1.11^a^	4.24 ± 1.12^a,b,c^	< 0.001
Hypertension (%)	3422 (42.3)	395 (58.0)^a^	1093 (74.1)^a,b^	460 (90.4)^a,b,c^	< 0.001
Diabetes (%)	639 (7.9)	88 (12.9)^a^	219 (14.8)^a^	99 (19.4)^a,b^	< 0.001
Obesity (%)	1083 (13.4)	82 (12.0)	499 (33.8)^a,b^	173 (34.0)^a,b^	< 0.001
History of cardiovascular disease (%)†	649 (8.0)	81 (11.9)^a^	217 (14.7)^a^	83 (16.3)^a^	< 0.001
Antihypertensive medication use (%)	747 (9.2)	124 (18.2)^a^	387 (26.2)^a,b^	230 (45.2)^a,b,c^	< 0.001
Antidiabetic medication use (%)	218 (2.7)	35 (5.1)^a^	77 (5.2)^a^	31 (6.1)^a^	< 0.001
LVIDD (cm)	4.68 ± 0.36	4.17 ± 0.33^a^	5.09 ± 0.47^a,b^	4.73 ± 0.40^a,b,c^	< 0.001
IVST (cm)	0.83 ± 0.08	0.95 ± 0.09^a^	1.03 ± 0.55^a,b^	1.15 ± 0.49^a,b,c^	< 0.001
PWT (cm)	0.82 ± 0.07	0.94 ± 0.08^a^	0.93 ± 0.09^a^	1.31 ± 1.06^a,b,c^	< 0.001
LVWT (cm)	1.65 ± 0.15	1.89 ± 0.17^a^	1.96 ± 0.56^a,b^	2.46 ± 1.20^a,b,c^	< 0.001
RWT	0.35 (0.33–0.38)	0.44 (0.43–0.46)^a^	0.37 (0.35–0.39)^a,b^	0.45 (0.43–0.48)^a,c^	< 0.001
LVM (g)	126.69 (109.44–142.71)	118.67 (109.69–147.83)	170.19 (147.78–200.40)^a,b^	193.96 (163.98–220.76)^a,b,c^	< 0.001
LVMI (g/m^2.7^)	34.89 (30.85–39.17)	36.44 (31.99–40.63)^a^	50.44 (47.25–56.24)^a,b^	54.34 (49.96–61.76)^a,b^	< 0.001
LVEDV (ml)	102.28 ± 18.66	77.88 ± 14.38^a^	124.62 ± 30.36^a,b^	105.15 ± 20.51^a,b,c^	< 0.001
LVESV (ml)	38.49 ± 12.06	31.14 ± 10.00^a^	47.76 ± 18.01^a,b^	40.52 ± 13.94^a,b,c^	< 0.001
LVEDVI (ml/m^2.7^)	27.94 ± 4.45	22.26 ± 3.45^a^	37.43 ± 8.51^a,b^	30.52 ± 4.84^a,b,c^	< 0.001
LVESVI (ml/m^2.7^)	10.51 ± 3.13	8.88 ± 2.67^a^	14.25 ± 5.09^a^	11.77 ± 3.81^a,b,c^	< 0.001
LVEF (%)	62.31 ± 9.72	59.83 ± 11.34^a^	61.87 ± 10.70^b^	61.65 ± 10.29^b^	< 0.001

Data are expressed as mean ± standard deviation or median (interquartile range) and numbers (proportion) as appropriate. CNY, China Yuan (1CNY = 0.158 USD); BMI, body mass index; TC, total cholesterol; TG, triglyceride; HDL-C, high-density lipoprotein cholesterol; LDL-C, low-density lipoprotein cholesterol; Non-HDL-C, non-high-density lipoprotein cholesterol; LV, left ventricular; LVIDD, left ventricular end-diastolic internal diameter; IVST, interventricular septal thickness; PWT, posterior wall thickness; LVWT, LV wall thickness; RWT, relative wall thickness; LVM, left ventricular mass; LVMI, left ventricular mass index; LVEDV, left ventricular end-diastolic volume; LVESV, left ventricular end-systolic volume LVEDVI, left ventricular end-diastolic volume index; LVESVI, left ventricular end-systolic volume index; LVEF, left ventricular ejection fraction; LVH, left ventricular hypertrophy

*P values are for an ANOVA or Kruskal-Wallis test (continuous) and chi-square test (categorical) comparison across left ventricular geometry

^a^*P* < 0.05 vs. normal geometry; ^b^*P* < 0.05 vs. concentric LV remodeling; ^c^*P* < 0.05 vs. eccentric LVH

†Including coronary heart disease, arrhythmia and heart failure

In unadjusted linear regression analyses, nontraditional lipid profiles were all positively correlated with larger LVEDVI and LVWT, with more modest effects on LVMI and concentricity index (Table 2). After multivariable adjustments for covariates, TC/HDL-C, LDL-C/HDL-C, and non-HDL-C remained as an independent determinant of concentric LVH phenotype, including greater LVMI (β = 0.036, 0.037, and 0.032, respectively), concentricity index (β = 0.027, 0.031, and 0.027, respectively), and LVWT (β = 0.048, 0.043, and 0.039, respectively; *P* < 0.01 for each). As such, greater LVMI, concentricity index and LVWT (β = 0.021, 0.020 and 0.043, *P* < 0.05 for both) response to increasing TG/HDL-C were also observed.

**Table 2:** Linear regression models of relation of nontraditional lipid profiles to LV structural parameters

Variables	LVMI		LVEDVI		Concentricity		LVWT	
	β	*P* value	β	*P* value	β	*P* value	β	*P* value
TC/HDL-C ratio								
Model 1	0.070	< 0.001	0.089	< 0.001	0.041	< 0.001	0.092	< 0.001
Model 2	0.036	< 0.001	0.026	0.009	0.027	0.007	0.048	< 0.001
TG/HDL-C ratio								
Model 1	0.057	< 0.001	0.073	< 0.001	0.036	< 0.001	0.091	< 0.001
Model 2	0.021	0.036	0.008	0.405	0.020	0.050	0.043	< 0.001
LDL-C/HDL-C ratio								
Model 1	0.073	< 0.001	0.085	< 0.001	0.046	< 0.001	0.090	< 0.001
Model 2	0.037	< 0.001	0.018	0.071	0.031	0.003	0.043	< 0.001
Non-HDL-C								
Model 1	0.071	< 0.001	0.076	< 0.001	0.047	< 0.001	0.086	< 0.001
Model 2	0.032	0.001	0.009	0.347	0.027	0.006	0.039	< 0.001

Models constructed with LV structural parameters as dependent variables and nontraditional lipid profiles as independent variables. β coefficient is per 1 SD increase of the nontraditional lipid profile. Abbreviations: *TC* total cholesterol, *TG* triglyceride, *LDL-C* low-density lipoprotein cholesterol, *HDL-C* high-density lipoprotein cholesterol, *Non-HDL-C* non-high-density lipoprotein cholesterol, *LVMI* left ventricular mass index, *LVEDVI* left ventricular end-diastolic volume index, *LVWT* left ventricular wall thickness. Model 1: unadjusted; Model 2: adjusted for age, sex, race, education level, family income, diet score, smoking and drinking status, physical activity, obesity, hypertension, diabetes, history of cardiovascular disease, antihypertensive medication use, and antidiabetic medication use

In analyses modeling nontraditional lipid profile as a continuous variable, we revealed a 15, 16, and 17% higher risk for concentric LVH with each SD increment in TC/HDL-C, LDL-C/HDL-C, and non-HDL-C levels, respectively (Table 3). After additional adjustment for the potential mediators (Model 2), TG/HDL-C carried concentric LVH odds (95% CIs) of 1.134 (1.030 to 1.249), per SD increase. In models incorporating TC/HDL-C tertiles, participants in highest TC/HDL-C tertiles experienced a 64% high risk of concentric LVH compared with the lowest group. Tertile analysis of the relationship between LDL-C and non-HDL-C and concentric LVH demonstrated a suggestion of excess risk with the highest tertile (fully adjusted OR: 1.523, 95% CI: 1.176–1.971; OR: 1.454, 95% CI: 1.121–1.888, respectively). Meanwhile, the corresponding risk comparing the top versus bottom tertile of TG/HDL-C was 1.260 (1.082–1.467) for eccentric LVH and 1.413 (1.097–1.821) for concentric LVH.

**Table 3:** Odds ratio of abnormal left ventricular geometric patterns according to continuous or tertiles of nontraditional lipid profiles

Variables	Concentric LV remodeling		Eccentric LVH		Concentric LVH	
	Model 1	Model 2	Model 1	Model 2	Model 1	Model 2
TC/HDL-C ratio (Per 1 SD increase)	0.998 (0.922–1.079)	0.973 (0.894–1.059)	1.261 (1.196–1.329)^a^	1.051 (0.990–1.116)	1.382 (1.276–1.496)^a^	1.150 (1.050–1.260)^b^
Tertile of TC/HDL-C ratio						
T1 (≤3.35)	1.000 (reference)	1.000 (reference)	1.000 (reference)	1.000 (reference)	1.000 (reference)	1.000 (reference)
T2 (3.35–4.29)	1.044 (0.863–1.264)	1.010 (0.832–1.226)	1.433 (1.242–1.653)^a^	1.183 (1.017–1.375)^c^	1.673 (1.300–2.152)^a^	1.463 (1.126–1.900)^b^
T3 (> 4.29)	1.084 (0.896–1.312)	1.032 (0.844–1.261)	1.829 (1.592–2.101)^a^	1.168 (1.003–1.361)^c^	2.593 (2.046–3.287)^a^	1.639 (1.268–2.120)^a^
P for trend	0.405	0.759	< 0.001	0.059	< 0.001	< 0.001
TG/HDL-C ratio (Per 1 SD increase)	1.055 (0.976–1.140)	1.047 (0.963–1.138)	1.278 (1.211–1.349)^a^	1.063 (0.999–1.131)	1.399 (1.285–1.522)^a^	1.134 (1.030–1.249)^c^
Tertile of TG/HDL-C ratio						
T1 (≤0.68)	1.000 (reference)	1.000 (reference)	1.000 (reference)	1.000 (reference)	1.000 (reference)	1.000 (reference)
T2 (0.68–1.28)	1.116 (0.920–1.352)	1.082 (0.890–1.315)	1.446 (1.252–1.670)^a^	1.171 (1.006–1.363)^c^	1.611 (1.259–2.062)^a^	1.295 (1.001–1.675)^c^
T3 (> 1.28)	1.194 (0.986–1.445)	1.162 (0.950–1.420)	1.966 (1.710–2.259)^a^	1.260 (1.082–1.467)^b^	2.381 (1.885–3.008)^a^	1.413 (1.097–1.821)^b^
P for trend	0.070	0.144	< 0.001	0.003	< 0.001	0.009
LDL-C/HDL-C ratio (Per 1 SD increase)	0.984 (0.909–1.064)	0.960 (0.882–1.045)	1.237 (1.174–1.304)^a^	1.014 (0.955–1.077)	1.404 (1.298–1.518)^a^	1.158 (1.059–1.266)^b^
Tertile of LDL-C/HDL-C ratio						
T1 (≤1.79)	1.000 (reference)	1.000 (reference)	1.000 (reference)	1.000 (reference)	1.000 (reference)	1.000 (reference)
T2 (1.79–2.44)	0.963 (0.794–1.166)	0.934 (0.768–1.135)	1.465 (1.272–1.688)^a^	1.131 (0.974–1.314)	1.686 (1.311–2.168)^a^	1.346 (1.035–1.750)^c^
T3 (> 2.44)	1.072 (0.888–1.295)	1.029 (0.844–1.255)	1.677 (1.459–1.927)^a^	1.001 (0.858–1.168)	2.534 (1.999–3.212)^a^	1.523 (1.176–1.971)^b^
P for trend	0.465	0.775	< 0.001	0.878	< 0.001	0.002
Non-HDL-C (Per 1 SD increase)	1.106 (1.026–1.192)^c^	1.045 (0.964–1.133)	1.260 (1.196–1.327)^a^	1.021 (0.963–1.084)	1.435 (1.329–1.549)^a^	1.174 (1.075–1.281)^a^
Tertile of Non-HDL-C						
Tertile 1 (≤3.33)	1.000 (reference)	1.000 (reference)	1.000 (reference)	1.000 (reference)	1.000 (reference)	1.000 (reference)
Tertile 2 (3.33–4.17)	1.111 (0.915–1.349)	1.024 (0.841–1.247)	1.447 (1.255–1.669)^a^	1.101 (0.947–1.280)	1.846 (1.431–2.381)^a^	1384 (1.063–1.802)^c^
Tertile 3 (> 4.17)	1.225 (1.009–1.486)	1.055 (0.861–1.292)	1.748 (1.518–2.012)^a^	1.020 (0.875–1.190)	2.623 (2.054–3.351)^a^	1.454 (1.121–1.888)^b^
P for trend	0.040	0.606	< 0.001	0.911	< 0.001	0.008

Abbreviations: *OR* odd ratio, *95% CI* 95% confidence interval, *TC* total cholesterol, *TG* triglyceride, *LDL-C* low-density lipoprotein cholesterol, *HDL-C* high-density lipoprotein cholesterol, *Non-HDL-C* non-high-density lipoprotein cholesterol, *LVH* left ventricular hypertrophy. Model 1: unadjusted; Model 2: adjusted for age, sex, race, education level, family income, diet score, smoking and drinking status, physical activity, obesity, hypertension, diabetes, history of cardiovascular disease, antihypertensive medication use, and antidiabetic medication use

^a^*P* < 0.001, ^b^*P* < 0.01, ^c^*P* < 0.05

In terms of discriminative value for prediction of morphologic LV abnormalities, as assessed by AUC, none of the other nontraditional lipid parameters were significantly better than TG/HDL-C in detecting eccentric LVH (AUC: 0.580, 95% CI: 0.571–0.589, optimal cutoff value: 0.58, sensitivity: 66.7% and specificity: 47.3%). The AUC of TG/HDL-C was statistically identical with TC/HDL-C and non-HDL-C, but was remarkably larger than LDL-C/HDL-C. The optimal predictive power for concentric LVH (AUC: 0.622, 95% CI: 0.613–0.631, sensitivity 72.1% and specificity 46.9%) was identified in non-HDL-C with a value of 3.42 or more, which was slightly stronger than TC/HDL-C (AUC: 0.605), TG/HDL-C (AUC: 0.601), and LDL-C/HDL-C (AUC: 0.613), despite the ROC curves presented near equivalence of performance for these four nontraditional lipid indices (Table 4).

**Table 4:** Area under the ROC curve (AUC) for identification of left ventricular geometric abnormalities by each nontraditional lipid index

Variables	AUC (95% CI)	*P* value	Cutoff point	Sensitivity (%)	Specificity (%)
Eccentric LVH					
TC/HDL-C ratio	0.574 (0.565–0.583)^a^	< 0.001	≥ 3.37	63.2	49.3
TG/HDL-C ratio	0.580 (0.571–0.589)^a^	< 0.001	≥ 0.58	66.7	47.3
LDL-C/HDL-C ratio	0.567 (0.558–0.576)	< 0.001	≥ 2.50	68.1	43.9
Non-HDL-C	0.570 (0.561–0.579)	< 0.001	≥ 3.51 mmol/L	60.2	51.1
Concentric LVH					
TC/HDL-C ratio	0.605 (0.595–0.614)	< 0.001	≥ 3.53	62.7	54.9
TG/HDL-C ratio	0.602 (0.593–0.611)	< 0.001	≥ 0.66	61.9	53.3
LDL-C/HDL-C ratio	0.613 (0.603–0.622)	< 0.001	≥ 2.71	66.0	51.9
Non-HDL-C	0.622 (0.613–0.631)	< 0.001	≥ 3.42 mmol/L	72.1	46.9

Abbreviations: *AUC* area under the ROC curve, *95% CI* 95% confidence interval, *TC* total cholesterol, *TG* triglyceride, *LDL-C* low-density lipoprotein cholesterol, *HDL-C* high-density lipoprotein cholesterol, *Non-HDL-C* non-high-density lipoprotein cholesterol, *LVH* left ventricular hypertrophy

^a^Indicates a significant difference as compared to LDL-C/HDL-C ratio

## Discussion

The constellation of our findings offered novel evidence for an independent positive association of nontraditional lipid profiles (TC/HDL-C, TG/HDL-C, LDL-C/HDL-C, and non-HDL-C) with concentric LVH, a hallmark of preclinical CVD, in the general population of rural China. That is to say, elevated nontraditional lipid parameters levels were associated with increased risk of concentric LVH in a dose-response fashion and subsequently could be used to monitor programs aimed at mitigating adverse structural remodeling of the heart and identifying individuals at high risk for CVD. Our study further suggested that nontraditional lipid profiles lowering might yield benefits in concentric LVH prevention.

Nontraditional lipid profiles has been shown in numerous studies to be useful screening tools for various populations, identifying those with higher risk of subsequent CV complications and heart failure [17–21]. As anticipated from previous work, TC/HDL-C ratio, a more accurate parameter reflecting the features of the discordance between particle concentration and cholesterol content [22, 23], was instrumental in identifying coronary atheroma progression and cardiovascular events, as well as provided atherogenic lipid particles information, especially when apolipoprotein fractions, LDL-C, and non-HDL-C levels were discordant [18, 20, 24, 37]. TG/HDL-C ratio has proved itself to be an effective marker of proatherogenic small and dense LDL particles [25, 26], and thus of insulin resistance and poor cardiometabolic profile. This increased concentration of high risk particles is predictive of an augmented risk of CHD in multiethnic populations [17, 19]. Further, atherogenic metabolic disorders evaluated in LDL-C/HDL-C ratio was an excellent indicator of coronary lipid-rich plaque and early stage atherosclerosis [18, 28, 38]. Finally, there was also some evidence that non–HDL-C, in comparison to LDL-C, was more relevant to atherogenic lipoprotein subfractions and might be a potentially better index in evaluating the cardiovascular risk [18, 20, 21].

The degree of contribution of dyslipidemia to LV remodeling has been debated, with evidence both for and against the predictive utility of lipid concentrations on indexes of LV structure [8–16]. Hence, the adoption of nontraditional lipid profiles to estimate altered LV morphology has fostered a growing enthusiasm. Proposals to replace information on classic lipid risk factors with lipid ratios or a single index were motivated by the idea that nontraditional lipid profiles better represent the underlying atherosclerotic process. In a very large population of Italian outpatient children with overweight, Di Bonito P and coworkers concluded that TG/HDL-C ratio discriminated better than non-HDL-C with prevalent concentric LVH [29, 39]. Supporting this finding, LDL-C/HDL-C contributed to the origin of LVH included in a prospective longitudinal cohort study over 20-year period [40]. In that study, the odds ratio for LVH was 1.31 (95% CI: 1.02–1.67) after controlling for history of ischemic heart disease, valvular disease, and use of antihypertensive medication. Partially different findings emerged from Framingham Heart Study, where there was no meaningful relation of changes in LV structure with non-HDL-C levels [11]. On the other hand, experimental evidence suggested that hypercholesterolemia was noted to have a harmful effect on cardiac systolic and diastolic function, and LV remodeling in response to myocardial infarction (MI) [41, 42]. Indeed, investigators from the Framingham Heart Study identified dyslipidemia (indicated by elevated non-HDL-C) was known to promote the development of heart failure, with mildly attenuated effect size by adjusting interim MI [43]. This notion was further supported by the Multiethnic Study of Atherosclerosis (MESA) study that found the coexistence of high TC/HDL-C ratio with diabetes aggravated a program of HF risk [44]. We speculated that these correlations were probably partially attributable to the possible association of nontraditional lipid profiles with the prevalence of adverse cardiac remodeling. Abnormal LV geometry has been systematically discussed in the context of incident HF and believed to be related to worse prognosis and outcomes. It is now becoming evident that LV geometric abnormalities have been recognized as a cumulative indicator that reflects the severity and chronicity of cardiovascular risk factors, which highlights the need to develop a better risk stratification and tailored screening tool. In this sense, this middle-aged, cross-sectional, population-based design is initiated to examine and validate the practicality of nontraditional lipid profiles as the key correlates of LV geometric abnormalities in rural China.

The present study strengthened and extended the conclusions of some previous investigations on the predictive impact of dyslipidemia on morphological alterations of LV geometry. After taking into account a number of confounders, TC/HDL-C and TG/HDL-C increased the risk for presence of concentric LVH, while the probability of eccentric LVH only modestly increased in the presence of high TC/HDL-C, and was mainly explained by metabolic abnormalities. Similarly, LDL-C/HDL-C and non-HDL-C were associated with a pronounced increase in concentric LVH. The relation of these lipid ratios to eccentric LVH became nonsignificant when we adjusted for hypertension, diabetes, obesity, history of CVD, antihypertensive and antidiabetic medication use, and we contemplated that its effects may partly be accountable to these conditions. These results were not in accord with the prior findings of Framingham Heart Study, potentially due to significant differences in the ethnicity and the age range of the studied population. Our conclusions may be clinically relevant because changes in LV geometry, specifically concentric LVH, have typically been appreciated as an important predictor of a greater risk of CV events.

There is a panel of plausible pathomechanisms responsible for the increased risk of abnormal LV geometry in those with poor nontraditional lipid profiles. HDL-C is regarded an important mediator of preventing myocardial lipid accumulation through reverse cholesterol transport, a process that involves elimination of free cholesterol by transferring and uptaking of free cholesterol from the peripheral tissues to the liver [45]. Inflammation has been a potential candidate for adverse LV remodeling [46], HDL-C exerted a protective effect by its anti-inflammatory properties [47]. Taken together, HDL-C was involved in improving endothelial function and weakening LDL oxidation, whereby protecting against cardiac and vascular remodeling [43]. These manifestations could be attributed to negative expression of cell surface adhesion molecules and inhibition of monocyte infiltration into the endothelium [48]. Secondly, under physiologic conditions of obesity and non-ischemic failing human hearts, increased stores of TGs were detectable in the myocardium [49]. The lipotoxic byproduct, generating from deleterious fatty acid pathways after myocardial TG overstorage entered a continuous cycle between hydrolysis and fatty acid esterification, resulted in the deterioration of cardiac function [50]. Thirdly, nontraditional lipid profile, such as TG/HDL-C, was a strong correlate of insulin resistance, which has been an important route leading to LVH [8–10]. Furthermore, excess cholesterol predisposes to vascular lesions, an early feature of arteries rigidity, which is a factor potentially to be the driving force behind increased LVM. Given the positive effect of systemic atherosclerosis on LVH, it is intriguing that relations were best described between nontraditional lipid indices and concentric LVH [51]. Thus, it is possible that abnormal LV geometry at that early stage of heart failure may be remediable to lipid-lowering therapy.

Our novel investigations have crucial clinical and public health implications for pointing out that those with high levels of nontraditional lipid profiles had a significantly greater burden of adverse LV remodeling that begins as subclinical changes in cardiac structure and eventually progresses to clinical HF. The present study included a large community-based sample with an available comprehensive panel of lipid measurements and echocardiographic data in Northeast China. The simplicity and cost-effective of nontraditional lipid profiles have made it possible for application to large-scale clinical and epidemiological studies and evaluated LV geometry and its modification over time to CVD outcomes. Despite the aforementioned strengths, certain limitations merit consideration. The cross-sectional design allows for only determining an association of nontraditional lipid profiles with concentric LVH, but fails to imply a causal relationship and track changes in lipid concentrations over time. Further confirmations in prospective studies to assess the prognostic role of nontraditional lipid profiles in LV morphology are warranted. In addition, although echocardiography has been well validated for measuring indices of cardiac structure, cardiovascular magnetic resonance (CMR) is thought to be highly accurate, reproducible, and widely considered the gold standard for the assessment of ventricular size and function. Finally, our results are predominantly applicable to Chinese adults, which limit generalization to the broader racial or ethnic populations.

## Conclusions

Nontraditional lipid profiles emerged as a valuable operational approach, a potential alternative to traditional lipid parameters, to refine risk stratification in individuals with concentric LVH. Under this scenario, these readily available lipid measurements could be applied as a relatively simple tool for subclinical signs of cardiac abnormalities assessment. The feasible benefit of aggressive primary prevention in combination lipid modifier therapy for adverse LV remodeling subjects with residual atherogenic dyslipidemia requires further investigation.

## Abbreviations

BMI: Body mass index
CVD: Cardiovascular disease
DBP: Diastolic blood pressure
FPG: Fasting plasma glucose
HDL-C: High-density lipoprotein cholesterol
IVST: Interventricular septal thickness
LDL-C: Low-density lipoprotein cholesterol
LV: Left ventricular
LVEDV: Left ventricular end-diastolic volume
LVEDVI: Left ventricular end-diastolic volume index
LVEF: Left ventricular ejection fraction
LVESV: Left ventricular end-systolic volume
LVESVI: Left ventricular end-systolic volume index
LVH: Left ventricular hypertrophy
LVIDD: Left ventricular end-diastolic internal diameter
LVM: Left ventricular mass
LVMI: Left ventricular mass index
LVWT: LV wall thickness
Non-HDL-C: Non-high-density lipoprotein cholesterol
PWT: Posterior wall thickness
RWT: Relative wall thickness
SBP: Systolic blood pressure
TC: Total cholesterol
TG: Triglyceride
